# Hygienic measures during animal transport to abattoirs - a status quo analysis of the current cleaning and disinfection of animal transporters in Germany

**DOI:** 10.1186/s40813-017-0078-x

**Published:** 2018-01-08

**Authors:** Luisa Weber, Diana Meemken

**Affiliations:** 1 Institute of Food Quality and Food Safety, University of Veterinary Medicine Hannover, Foundation, Bischofsholer Damm 15, 30173 Hannover, Germany; 2 Institute of Food Safety and Food Hygiene, Freie Universitaet Berlin, Koenigsweg 67, 14163 Berlin, Germany

**Keywords:** Animal transport, Cleaning, Disinfection, Animal disease

## Abstract

**Background:**

The process of cleaning and disinfection of animal transport vehicles after unloading animals at the abattoir is a critical control point regarding proper hygiene. It is an important step regarding the biosecurity. In the present study, a status quo analysis of the currently performed cleaning and disinfection measures of animal transport vehicles was carried out at the vehicle washing facilities of five different industrial abattoirs in Germany. For this purpose, a checklist was developed and validated to assess the washing procedure of transport vehicles in a standardised way. The evaluated phases of cleaning included the evaluation criteria “length of time per used floor”, “visual cleaning success” and the “hygienic awareness of the driver”. During disinfection, attention was paid to the internal and external surfaces of the transporter and to the methods used to disinfect them. In addition, the technical and structural equipment of the five different washing facilities were recorded using a questionnaire and compared to the legal regulations, respectively. At each location, approximately 150 vehicles of all delivery types (transport vehicles owned by the abattoir, external delivery companies and vehicles owned by the supplying farmers) were inspected so that in total a number of more than 750 vehicles were included in this study. The aim was to develop abattoir specific, as well as generally applicable intervention measures and to generate “standard-operation procedures” (SOP’s) for the cleaning and disinfection of animal transporters.

**Results:**

At two out of five locations vehicles have left the abattoir without cleaning and disinfection. In 31–97% of all vehicles, only a cleaning of the vehicle was carried out, a subsequent disinfection did not take place. A cleaning followed by disinfecting took place in only 3–59% of all vehicles.

**Conclusion:**

The results indicate a considerable need for improvement and standardisation in this relevant field of disease prevention.

## Background

The cleaning and disinfection process is an important step in animal disease prevention and is in the responsibility of the respective animal transport drivers. If this is not carried out, the biosecurity of livestock can be put at risk by a potential contamination and consequently by a spread of pathological agents to farms [1]. The statutory requirements are stipulated in the German Cattle Transport Ordinance. In section 17 thereof it states that cattle trailers which are transported to cattle loading sites, collection points or abattoirs must be cleaned and disinfected before leaving the premises again [2]. In accordance with the European Regulation (EU) No 853/2004, abattoirs must have “a separate place with suitable facilities for the cleaning, washing and disinfection of transport equipment for animals”. Detailed explanations as to how the cleaning and disinfection has to be carried out or under which conditions it has to take place are not described in detail. This highly sensitive and very important task is also often underestimated in animal disease prevention approaches because of these “legal gaps” or undefined procedures. It is with high importance for all endemic diseases and a general rule for a high biosecurity. But also with regard to the current animal disease situation, our research project focused on the animal disease African swine fever. In the affected wild boar populations in Eastern Europe (Poland and the Baltic states) African swine fever is spreading further [3]. Due to this current trend, a western spread seems likely. Many non-EU member states including Belarus, Ukraine, Armenia and Azerbaijan also reported cases of African swine fever [3]. So far, little has been known concerning the extent of transport and waste disinfection [4]. Therefore, questions arise whether, and above all, how the cleaning and disinfection are carried out by the responsible persons and what conditions occur in the washing areas.

## Methods

The investigations were carried out by one and the same veterinarian at five different industrial abattoirs in the federal state of North Rhine-Westphalia in Germany. The period of data collection lasted from June to October 2016. The observation period lasted from about 6.00 a.m. to about 4.00 p.m., from Monday to Saturday. On the basis of visual controls, the entire process of cleaning and disinfection was evaluated in a standardised manner. Therefore, the veterinarian used checklists, which had been prepared and validated in a pilot-study to previously determined assessments of relevance and importance (see attachment). Approximately 150 vehicles per location, in total 756 vehicles, were evaluated. Both cattle as well as pig transports were included. The transports took place all within Germany. The cattles were transported on single deck or double-deck trucks, the pigs on single-to three-deck trucks. On average the surface area of such trucks is 41m^2^ and of trailers 46m^2^. In comparison to that, the average transport area of trailers pulled by cars is 7m^2^ and of trailers pulled by tractors 36 m^2^ [5]. Among other parameters, the time period of the cleaning and disinfection process was measured. Moreover, the quality of the different procedures was assessed by visual controls. The main focus was placed on the aspects that may enable a spread of pathogens: Both the inner surface of the transport vehicle and the entire outer side, including the tyres and wheel arches as well as the vehicle underside being observed. In addition to the actual driver’s behaviour, the various washing facilities (*n* = 5) were analysed at the different locations. In particular, the local conditions and the technical equipment and functionality were examined and assessed by means of an additional questionnaire. Since there are hardly any legal regulations existing for this purpose, various expert opinions were sought to provide optimal equipment for the livestock truck washing facilities with minimum requirements in future. Furthermore, the aim of the study was to compare the given possibilities with the legal situation as well as with those recommendations of the experts and those presented in our study. The data were analysed descriptively (Microsoft Excel, 2013) and by means of the statistical programme SAS Enterprise Guide 7.1, SAS Institute Inc., Cary, USA.

## Results

In total, the cleaning and disinfection process of 756 vehicles was assessed, split into sub-contracted agents and private transporters. The sum of vehicles owned by sub-contracted companies was split into internal freight companies and external freight companies. The private transporters were split into private cars pulling a trailer and tractors pulling a trailer. Both cattle as well as pig transports were rated (see Table 1). The evaluations were carried out in a comparative manner to assess the factors of influence concerning the equipment and functionality of the washing place.

**Table 1:** Sample size of analysis ordered according to location and vehicle type

Vehicle types	Location 1	Location 2	Location 3	Location 4	Location 5	In total
Internal freight forwarders	9	6	5	18	0	38 (5%)
External freight forwarders	94	83	143	106	147	573 (76%)
Private transport by car	17	35	4	4	1	61 (8%)
Private transport by tractor	29	27	0	24	4	84 (11%)
In total	149 (20%)	151 (20%)	152 (20%)	152 (20%)	152 (20%)	756

### The locations

The surveys were conducted at five industrial abattoirs, at which cattle and pigs or only cattle or only pigs were slaughtered. As shown in Table 2 these locations partly differ with regard to the equipment at the car wash. A high pressure cleaner with warm water was not available at any location. The washing process was started by inserting special coins or plastic keys. Different washing units of two or 5 min could be booked. Only one location charged for the washing according to the number of transported animals (flat-rate price). Once the driver had inserted a coin/plastic keys into the machine the unit was paid for and the time started. Usually the drivers had to estimate the time required and pay for the whole unit no matter how long they actually needed to clean their trailer. The decision rested with the driver’s, whether or not the units were then reloaded or the washing process ended, even when the cleaning process had not yet been completed. In some cases, a complete disinfection of the vehicle was not possible since the lance was not optimally positioned and thus was insufficient in terms of length. On several days, at location 1 5 days in total, at location 3 9 days in total, disinfection was either in one place or not possible at all. Either the equipment was defective because of missing nozzles, missing/defective lances or the disinfectant was empty. The consumption of disinfection was regularly recorded at location 2, whereas this was not monitored at any of the other locations. The cleaning and disinfection were carried out at the same place. A “one-way street” system (no separate “dirty” entrance or “clean” exit) was not available at any location. At location 1 the cleaning of the complete vehicle in one step (tractor and trailer) was not possible because the space was too limited, here the tractor and the trailer had to be cleaned and disinfected separately. At locations 1, 2 and 4, the car wash streets could be entered from both sides, at location 3 from one side and at location 5, the vehicles had to reserve into the washing places. For the personal hygiene of the drivers, there were washing facilities for hands and/or footwear at three locations. Two locations offered no washing facilities for the drivers at all.

**Table 2:** Overview of the abattoirs (Location 1–5) with the different washing facilities

Equipment	Location 1	Location 2	Location 3	Location 4	Location 5
Slaughtering	Cattle and pigs	Cattle; Collection point for pigs	Pigs	Pigs	Pigs
Slaughters per year	Approx. 1.260.000 (pigs) Approx. 57.000 (cattles)	Approx. 145.000	Approx. 457.500	Approx. 2.281.000	Approx. 2.050.500
Vehicles being washed per day	65–70	25–40	25–40	80–90	30–40
No. of washing bays	6 (including 2 for internal carrier)	4	3	5	4
Driveable with complete length	No	Partially, depending on vehicle length	Yes	No	Yes
Enclosure	No	Yes, without any door systems	No	Yes, with a sliding door	No
Side walls/Separation	No	Yes, but they didn’t cover the entire length	No	No	No
Hose reel	Manual	Manual	Partially with automatic pipe-winder	Manual	Pipe- winder
Activation	Wash-keys	Coins	Wash-keys	Wash-keys	Loaded no. of units was paid for
Cleaning	58 L/min 11–12 bar cold water	100 L/min 10 bar cold water	Not specified cold water	142 L/ min cold water	174 L/ min 10 bar cold water
Disinfection	1 lance for 2 washing places) hose sometimes too short to reach everywhere	1 lance for 4 washing places; hose sometimes too short to reach everywhere	1 lance for 3 washing places; hose sometimes too short to reach everywhere	1 lance for 2 washing places	1 lance for 2 washing places; hose sometimes too short to reach everywhere
Additional disinfecting options	Mobile disinfection basin	Disinfection basin shortly before exit (only in disease cases)	Washing place can be converted into “drive-through” disinfection basin	Mobile disinfection basin	Bridge underpass can be converted into a “drive-through” disinfection basin (only in disease cases)
Cleaning and disinfection for hands and footwear	Hand washing is possible	Hand washing is possible	Unavailable	Hand and footwear washing is possible	Unavailable
Others	Driveable from two sides heated flooring (at the washing place)	Cleaning agent (1 hose for 4 places)	Some hoses have to be brought along; nozzles have to be brought along	Nozzles have to be brought along	Have to reverse into it; Manure tray is directly adjacent to the washing places

### The cleaning and disinfection process

**Fig. 1:**
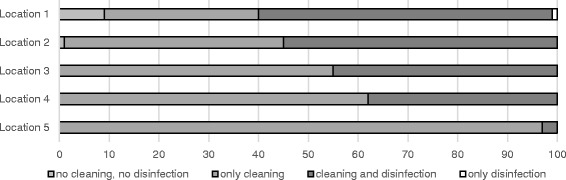
Performed cleaning and disinfection of all vehicles at the different locations in percent

**Fig. 2:**
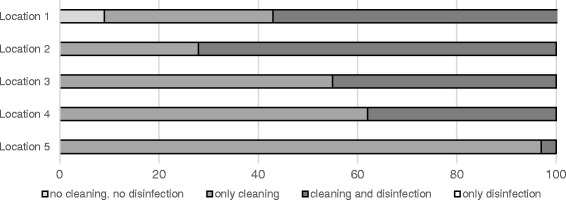
Cleaning and disinfection of internal and external freight forwarders at the different locations in percent

**Fig. 3:**
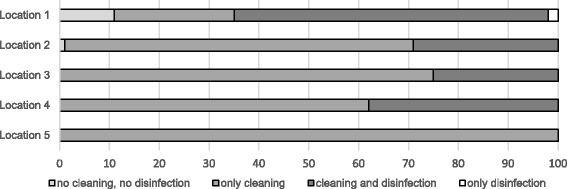
Performed cleaning and disinfection of private transports at the different locations in percent

**Fig. 4:**
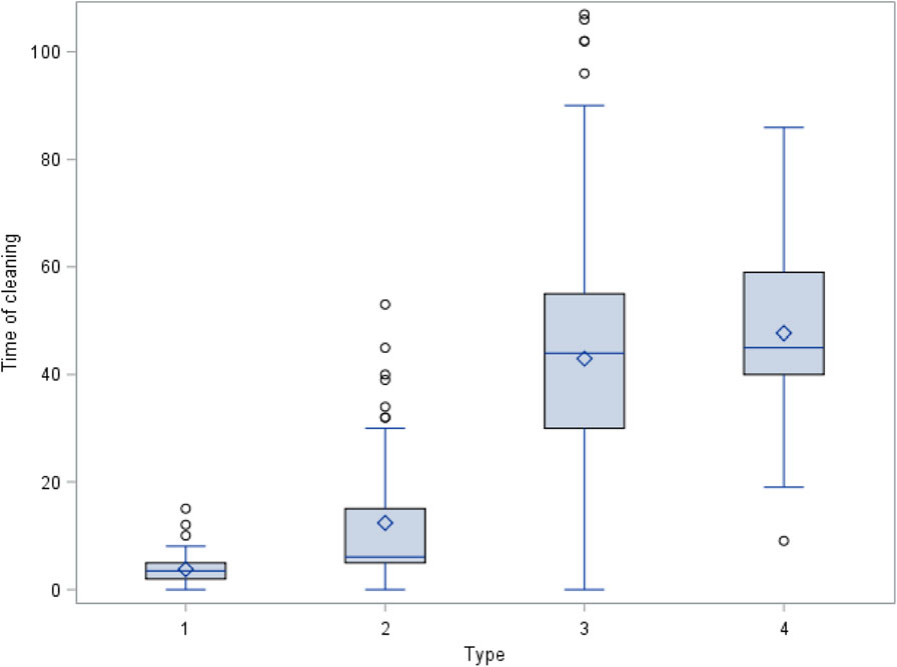
Boxplot of the time of cleaning, grouped by type 1: Private transport by car, 2: Private transport by tractor, 3: External freight forwarders, 4: Internal freight forwarders

**Fig. 5:**
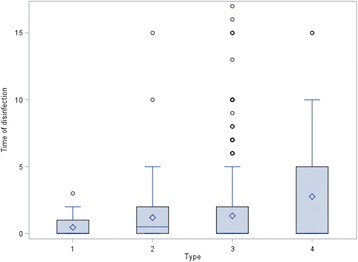
Boxplot of time of disinfection, grouped by type 1: Private transport by car, 2: Private transport by tractor, 3: External freight forwarders, 4: Internal freight forwarders

At two locations some vehicles left the abattoir without any cleaning and disinfection at all. Furthermore, at location 2, 1% of all vehicles left with only superficially performed disinfection (Fig. 1**)**. The percentage of vehicles that were cleaned but not disinfected ranged from 31% (location 1) to 97% (location 5) (Fig. 1). The comparison between the truck companies and the private suppliers shows that cleaning with subsequent disinfection was carried out more frequently on a percentage basis by the drivers from the truck companies than by the private suppliers (Figs. 2 and 3). However, the amount of cleaning and disinfection does not necessarily mean that the two processes are of a high quality as shown in Tables 3, 4 and 5. Assessed by visual inspection, the cleaning process including removal of coarse dirt and the intensive cleaning of the surfaces and separating screens was carried out sufficiently well by 79% of the external and internal forwarding companies and by 31% of the private suppliers only. In only 11% of the vehicles of the internal and external truck companies and 36% of the private deliverers was the performance moderate. An insufficient performance was observed in 3% of the internal and external truck companies and in 19% of the private suppliers (Table 4). At the end of the cleaning process some of the heavy-duty dirt and strongly adhering dirt were still visible. In these cases, it was necessary to assume that no thorough cleaning had taken place. The average cleaning time for the internal and external forwarding companies was 8.5 min per used floor and 6 min per used floor for the private suppliers with car/tractor-drawn trailers (Fig. 4). In the case of the external truck companies, drivers who had not cleaned and disinfected their vehicle at all (location 1), as well as those who needed a cleaning time of 107 min were included in our study. On average, the cleaning time for the external truck companies was 43 min (SD 19.2, *n* = 579) (Fig. 4). In comparison to these findings, all drivers of the internal forwarding company cleaned their vehicle. The minimum cleaning time for the entire vehicle was 9 min, the maximum 86 min with a mean of 48 min (SD 16.6, *n* = 37). Concerning the private suppliers, we recorded drivers who neither cleaned nor disinfected their vehicle at all (locations 1 and 3; cars as well as tractors pulling trailers). The maximum cleaning time for car-drawn trailers was 15 min and 53 min for the trailers pulled by tractors (Fig. 4). The mean duration was 4 min (SD 2.7, *n* = 63) for the car-drawn trailers and 12 min (SD 11.5, *n* = 77) for the tractor-drawn trailers (Fig. 4). Here it was striking that the car/tractor was only partially cleaned by 19% of the private suppliers (both tractors and car-trailers) and that a complete cleaning was carried out only by 7% of them. The rest cleaned neither the car nor the tractor, only the trailers. The parts of the vehicles which were previously considered to be critical with regard to possible contamination e.g. tixes and wheel arches were cleaned by 31% of the internal and external forwarding companies and by 11% of the private suppliers. Exclusive cleaning of the tires was carried out by 91% of internal and external truck companies and by 56% of private suppliers. The mean time needed for disinfection a single floor amounted to 16 s for the internal and external truck companies and 36 s for the private suppliers with car/tractor-towed trailers. At all locations drivers from each delivery type were recorded, who did not disinfect their vehicle at all. The maximum disinfection time ranged from3 min for the car-pulled trailers, 15 min for the tractor-pulled trailers and the internal haulage company to 17 min for the external truck companies (Fig. 5). On average, it took 0.5 min for the trailers pulled by private cars, 1 min for trailers pulled by tractors, 3 min for the internal company and 1.5 min for the external truck companies to disinfect their vehicles (Fig. 5). In many cases (86% of the internal and external truck companies, 42% of the private suppliers), disinfection was carried out starting from the outside into the inside of the vehicle. The disinfection lance was transported through the side windows or through a side flap into the vehicle (Table 5).

**Table 3:** Proportion of vehicle areas involved in cleaning from private transports and internal and external freight forwarders (in percent)

Area of vehicle	Private transport	Internal and external freight forwarders
Tyres and wheel cases	11	31
Only tyres	56	91
Underside of vehicle	14	30
Car/tractor partially	19	–
Car/tractor completely	7	–
Driver’s cab completely	–	33
Tailboards	77	97

**Table 4:** Quality of implementation from private transports and internal and external freight forwarders (in percent)

Implementation	Private transport	Internal and external freight forwarders
Good performance	31	79
Good-moderate performance	14	7
Moderate performance	36	11
Bad performance	19	3
Own brush	4	13
Own hose	1	6
Several add-ons (e.g. brush)	0	4

**Table 5:** Proportion of vehicle areas involved in disinfection from private transports and internal and external freight forwarders (in percent)

Area of the vehicle	Private transport	Internal and external freight forwarders
Only trailer	88	–
Trailer and car	7	–
Through the flaps	17	33
Through side window	25	52
No floor	22	10
All floors	66	31
Partition walls	66	88
Only tyres	32	24
Tyres and wheel cases	2	12
Driver’s cab	–	3
Tailboards	68	49
Exterior	27	25

## Discussion

In this study, the current situation regarding the cleaning and disinfection of animal transport vehicles at abattoirs was investigated. This process requires disease-control time-scale which should be carried out with the utmost priority and accurateness. Especially because of the current epidemic situation according to European experts’ opinion with regard to a potential introduction of African swine fever to Germany via Romania, Bulgaria or Hungary, there is the need for action. However, cleaning and disinfection of transporters is also an important procedure regarding general biosecurity and to prevent the onset of endemic diseases. Particularly in the case of animal diseases, the share of the transmission path by passenger and vehicle traffic was reported to amount to 20% [6]. The transport of animals constitutes a possible source of entry of these diseases. The topic of transport hygiene is also being discussed internationally concerning the possible spread of human pathogens via animal transport vehicles and the inadequate cleaning and disinfection thereof [4]. Although some legal requirements do exist at European level [7] and at national level [2], these legal regulations are insufficient to achieve an optimal standardised monitoring and control. The statutory requirements are stipulated in the German Cattle Transport Ordinance. In section 17 thereof it states that cattle trailers which are transported to cattle loading sites, collection points or abattoirs must be cleaned and disinfected before leaving the premises again [2]. In accordance with the European Regulation (EU) No 853/2004, abattoirs must have “a separate place with suitable facilities for the cleaning, washing and disinfecting of transport equipment for animals”. Detailed explanations as to how the cleaning and disinfection has to be carried out or under which conditions it has to take place are not described in detail. This highly sensitive and very important task is also often underestimated in animal disease prevention approaches because of these “legal gaps” or undefined procedures. The importance of cleaning and disinfection was realised for livestock collection centres, livestock companies and transport companies [2] and legal guidelines stipulated. However, provisions for the washing place equipment at abattoirs should be defined more clearly. In accordance with these existing guidelines both, cattle trading companies and hauliers as well as cattle collection points must have a suitable place for the cleaning and disinfection with pressurised warm water. However, the requirement temperature is not specified. In addition, the option is given for a third party to carry out the cleaning and disinfection process. It must be ensured that yearlong disinfection is carried out. The floor of the washing place has to be impervious to liquids and slope towards a drain for collection the wastewater. Furthermore, facilities must be provided for washing hands and footwear. Abattoirs are currently excluded from this existing legislation. Thus, these measures clearly have to be reviewed in order to achieve the best possible hygienic standards by optimising the existing legal requirements.

Another important focus of this study was the discussions with the drivers and the experts. The named reasons for a short cleaning time were, amongst other things, time problems, lack of technical equipment and the argument that a subsequent more intensive cleaning would be carried out later. Another issue was the use of washing tokens When the last unit was finished, even though little areas on the exterior surface had not been properly cleaned, this minor contamination was accepted rather than booking another unit even if the entire time were not used. In this case it would be better to bill the actual time taken and not the total number of units. Identified reasons for inadequate disinfection were defective/non-functioning technical equipment and time problems. Mentioned reasons were time problems, that the prescribed application time of the disinfectant could not be adhered to due to the subsequent planned tours, corrosive effects, missing user protection when the disinfectant was applied (for example, gloves, protective googles or the like), possible influence on the charging behaviour of the animals after disinfection and that only own animals were transported (private transport). Based on these and on our own experiences at the abattoirs as well as expert discussions, achievable objectives for livestock transport were defined and specific action plans were drawn up for each location. Only through this approach, certain standards can be implemented. The following aspects have crystallized which minimum requirements should fulfill. The actual situation was compared to the target situation, the legal requirements were listed and the measures to be initiated were set forth and prioritised. Among others, it was shown that minimum requirements include a complete enclosure of the washing space with roller door systems to be independent of any inclement weather, an adequate partition as well as a one-way street system to prevent possible recontamination. In order to achieve the best possible cleaning results, a low-pressure system (approx. 10 bar, water capacity 80–100 L/min) should be installed to rinse the trailer and remove all coarse debris. If necessary, a high-pressure cleaner with a minimum water temperature of 50 °C and ordinary cleaner should be used in a further step. The disinfection should be applied as a foam to ensure a longer time for it to take effect. Moreover, it adheres longer to the surfaces and reaches those areas where the disinfectant cannot be applied. Not only is the cleanliness of the trailers important but also the hygiene of the drivers. Therefore, there should at least be a possibility to wash one’s hands (soap, paper towels and disinfectant being available) as well as a separate device for cleaning and disinfection footwear. The user protection of the disinfectant must be taken into account: At least one protective suit (overalls or similar), gloves and, depending on the means used, protective goggles and a respiratory mask should be offered and used. In addition, an eye shower should be on site. An important aspect is that all objects and accessories to be used must be directly accessible to the driver. This is because our observations showed that only devices located directly at the washing facilities are accepted and used by the drivers. Random checks should be carried out regularly to monitor and to record the cleaning and disinfection processes, for example by means of adhesive-film tests. In addition, for the cleaning check and disinfection process, internal samples should be taken.

This study is based on visual inspections only; no microbiological samples were taken because the processes per se were more important than determining the exact bacterial cell count. However, after implementing the improved measures, ascertaining the total bacterial cell count should be carried out to regularly monitor the cleaning and disinfection processes. We recommend that it should be established as a standard monitoring measure in future. The present analysis illustrates the necessity for revising the requirements concerning equipment and handling. Furthermore, cleaning and disinfection at the livestock washing places must be introduced, especially in consideration of today’s prevalence of animal diseases. Moreover, the latter demonstrates the need for explicit legal requirements stipulating that companies must adhere to these since the study indicates, that adequate disinfection is not achievable under the existing conditions. In addition, the control function of veterinarians is essential in this sector. Without the presence and cooperation of veterinarians, it cannot be monitored whether the legal requirements are properly adhered to or not. At present, there is an urgent need for action to improve the prevention of animal diseases by optimising the execution of preventive measures. The cleaning and disinfection of transport vehicles is one of the most important steps towards achieving this.

## Conclusion

The cleaning and disinfection of animal transport vehicles is an important and not to be ignored factor in animal disease prevention. Only the combination of a legal requirements and a veterinary control can ensure a high quality standard of these processes. Also there is an urgent need for action and the awareness of all involved parties must be increased. A hygienic livestock transport helps to prevent the spread of endemic animal infections.

## Abbreviations

a.m.: Ante meridiem
approx.: Approximately
e.g.: Exempli gratia
EU: European Union
Fig: Figure
Figs: Figures
L/minute: Liters per minute
m^2^: Square meters
n, No: Number
p.m.: Post meridiem
SD: Standard deviation
SOP’s: Standard-operation procedures
